# Biportal Endoscopic Spine Surgery: Evolution of Techniques, Indications, and Influential Literature

**DOI:** 10.3390/jcm15051843

**Published:** 2026-02-28

**Authors:** Kareem S. Mohamed, Mark Kurapatti, Ethan Yang, Husni Alasadi, Wasil Ahmed, Ryan A. Lamidi, Suhas K. Etigunta, Akiro H. Duey, Bashar Zaidat, Brian H. Cho, Daniel C. Berman, Joshua Lee, Junho Song, Samuel K. Cho

**Affiliations:** 1 Department of Orthopaedic Surgery, Icahn School of Medicine at Mount Sinai, New York, NY 10029, USA; kareem.mohamed@icahn.mssm.edu (K.S.M.); mark.kurapatti@icahn.mssm.edu (M.K.); husnialasadi1@gmail.com (H.A.); wasil.ahmed@icahn.mssm.edu (W.A.); ryanlamidi25@gmail.com (R.A.L.); suhas.etigunta@icahn.mssm.edu (S.K.E.); akiro.duey@mountsinai.org (A.H.D.); basharzaidat@gmail.com (B.Z.); brian.cho2@mountsinai.org (B.H.C.); dcberman627@gmail.com (D.C.B.); joshua.h.lee4@gmail.com (J.L.); samuel.cho@mountsinai.org (S.K.C.); 2 Department of Orthopaedics, University of Maryland Medical Center, Baltimore, MD 21201, USA; ethan.yang@som.umaryland.edu

**Keywords:** biportal endoscopic spine surgery, unilateral biportal endoscopy, minimally invasive spine surgery, scoping review, spinal decompression, lumbar spine

## Abstract

Biportal endoscopic (BE) spine surgery has gained increasing attention as a minimally invasive alternative to conventional spinal procedures, yet the distribution of procedural applications and anatomic targets within influential BE-specific publications has not been clearly synthesized. This study aimed to synthesize influential publications on BE spine surgery to describe the evolution of procedural applications, anatomic focus, and clinically relevant themes reflected in the literature. A comprehensive search of the Web of Science database was performed using terms related to biportal and multiportal endoscopic spine techniques. Influential articles were identified using citation frequency as a screening criterion, and relevant study characteristics, including publication year, authorship, institutional affiliation, geographic region, journal, and spinal region addressed, were extracted. Full-text screening confirmed inclusion of true biportal endoscopic spinal procedures and categorized the anatomical region and surgical technique addressed. Publications spanned 1997 to 2023, with a marked increase after 2018 and peak productivity in 2022. Influential publications were most frequently published in World Neurosurgery, with substantial contributions originating from South Korea, including work by Dae-Jung Choi. Most studies focused on lumbar procedures, primarily decompression techniques and transforaminal lumbar interbody fusion. Overall, this review highlights the rapid clinical growth of BE spine surgery, with influential literature emphasizing lumbar applications and underscoring the need for further research on outcomes, learning curves, and broader international adoption.

## 1. Introduction

Lumbar disc herniation (LDH) and spinal stenosis are common conditions among individuals aged 60 and older, primarily due to degenerative changes associated with aging [1,2]. Traditional open surgeries, such as open laminectomy for spinal stenosis, can lead to paraspinal muscle atrophy caused by ischemia, denervation, and direct muscle trauma during dissection and retraction [3]. Moreover, these procedures carry risks of complications, including infection, prolonged hospital stays, postoperative pain, and increased blood loss [4,5]. In response to these challenges, the advent of minimally invasive spine surgery, particularly biportal endoscopic spinal surgery, promises potential significant progress and improved patient outcomes [6].

Biportal endoscopy (BE), also referred to as biportal endoscopic spinal surgery (BESS) and unilateral biportal endoscopy (UBE), employs two small portals—one for the endoscope and another for surgical instruments. This setup provides a magnified view of the surgical site, enabling precise execution of the procedure [7]. Proposed indications for BE include LDH, central lumbar, foraminal, and lateral recess stenosis, and other spinal conditions [8]. Compared to open surgery, BE offers several advantages, such as reduced perioperative bleeding, prevention of paraspinal muscle atrophy, and decreased perioperative pain [8,9]. Additionally, endoscopic spinal surgery is more cost-effective, with shorter hospital stays, quicker recovery periods, and reduced reliance on opioid pain medications [10]. Consequently, the number of endoscopic spinal surgeries performed in the U.S. has been steadily increasing, reflecting the growing recognition and adoption of these advanced techniques within the medical community [11].

Given the rapid emergence of biportal endoscopic spine surgery and its increasing use in clinical practice, a focused synthesis of the literature specific to this technique is warranted. While prior reviews have examined endoscopic spine surgery broadly, the distribution of procedural applications and anatomic targets within influential biportal-specific publications has not been clearly synthesized. This study was designed as a citation-informed scoping review of influential biportal endoscopic spine surgery publications in order to map the evolution of procedural applications and spinal regions addressed. By examining how biportal techniques have been applied across operative contexts, this review aims to contextualize current practice patterns and identify areas where further clinically oriented research is needed.

## 2. Methods

This scoping review was conducted in accordance with the PRISMA extension for scoping reviews (PRISMA-ScR). The Web of Science platform aggregates peer-reviewed publications across multiple indexed databases and disciplines.

A search was conducted using all available Web of Science databases (Web of Science Core Collection, BIOSIS Citation Index, Current Contents Connect, Data Citation Index, Derwent Innovations Index, Grants Index, KCI-Korean Journal Database, MEDLINE, and Preprint Citation Index). The Boolean search query was: (biportal OR bi-portal OR multiportal OR multi-portal OR “two portal”) AND (spin* NEAR/3 endoscop*).

As of 9 July 2025, the search yielded 235 records. Records were ranked in descending order by citation count. No fixed citation threshold was applied; instead, articles were reviewed in order of citation frequency to identify the most frequently cited publications meeting inclusion criteria. Citation frequency was used to identify publications with measurable impact within the biportal literature and was not intended as a proxy for methodological quality or level of evidence.

The 105 most-cited records underwent full-text assessment according to predefined eligibility criteria. Full-text review was performed independently by two authors, with discrepancies resolved by a third reviewer. Five articles were excluded after full-text review because they did not focus on a two-portal/biportal endoscopic spinal technique, resulting in 100 studies included in the final analysis.

**Figure 1:**
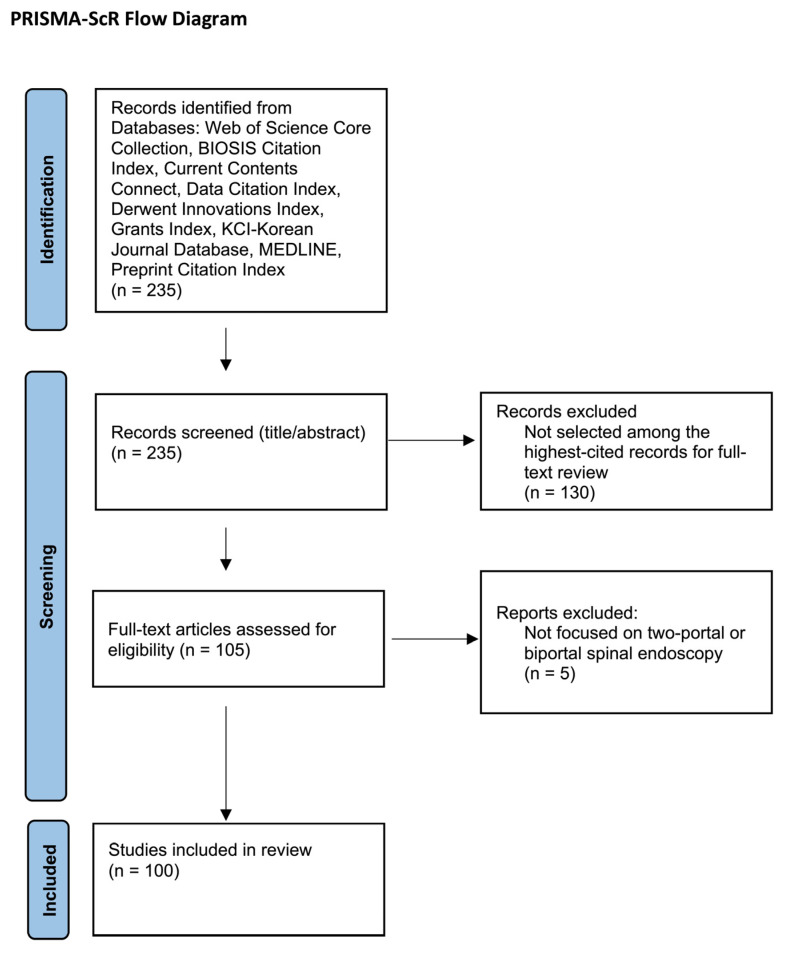
PRISMA-ScR flow diagram of study selection.

The inclusion criteria were: (1) full-text studies, (2) studies in English, and (3) studies discussing a biportal endoscopic technique in the context of spine surgery. For the purposes of this review, we considered ‘biportal endoscopic spine surgery (BESS)’, ‘unilateral biportal endoscopy (UBE)’, and ‘two-portal endoscopic spine surgery’ to be interchangeable terms describing a technique that utilizes two independent portals—one primarily for endoscopic visualization and one for instrumentation—applied to spinal pathology. Papers describing uniportal full-endoscopic techniques, tubular microscopic approaches, or other minimally invasive procedures without a two-portal endoscopic configuration were excluded. A PRISMA-ScR flow diagram detailing study identification, screening, eligibility assessment, and inclusion is provided in Figure 1. Figures were generated using R version 4.3.1. 

Data from the included influential articles were summarized using descriptive analysis tools within Web of Science. Extracted variables included publication year, authorship, publication venue, institutional affiliation, and geographic region. In addition to total citation counts, we calculated citations per year since publication to partially normalize for differences in article age. Manual data extraction was performed for studies where Web of Science’s analysis feature did not provide the necessary data. A full-text review was conducted to determine the application of biportal endoscopic techniques to specific spinal regions. The articles were then tagged by the type of application used to address the spinal pathology, which included techniques such as foraminotomy, laminotomy, transforaminal lumbar interbody fusion, etc.

## 3. Results

One of the earliest and most frequently referenced publications addressing biportal endoscopic spine surgery was “Fully endoscopic lumbar interbody fusion using a percutaneous unilateral biportal endoscopic technique: technical note and preliminary clinical results” by Heo et al. [12] (Table 1). Publication dates ranged from December 1997 to March 2023.

**Figure 2:**
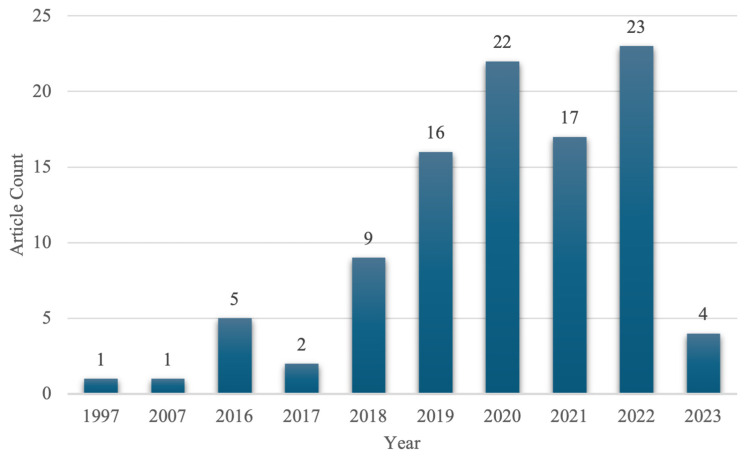
Temporal distribution of influential papers in biportal endoscopic spine surgery.

Included publications were distributed across multiple publication years (Figure 2). An increase in publication frequency was observed beginning in 2018. The highest number of included publications occurred in 2022. A similarly high number of publications occurred in 2020.

Because many studies involved authors with multiple institutional affiliations, a total of 290 institutional records were identified. Medical schools were excluded if their parent institutions were also recorded. Institutions represented in five or more included publications are summarized in Table 2. Yonsei University was represented in 12 publications. Chungnam National University and Barun Hospital were each represented in 11 publications.

**Figure 3:**
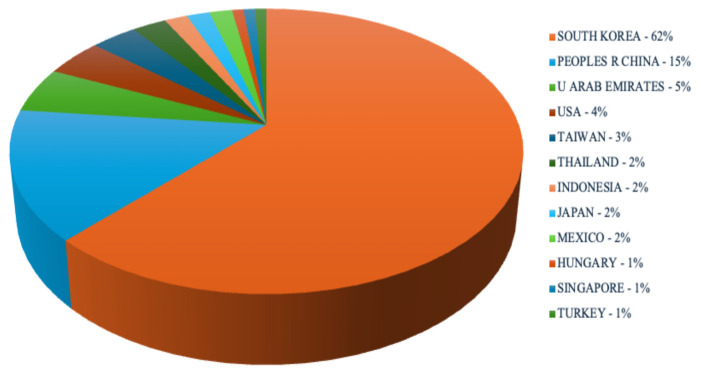
Countries contributing to influential papers in biportal endoscopic spine surgery.

Country of origin was recorded for all included publications. Because several studies involved international collaboration, a total of 116 country records were identified (Figure 3). Publications originating from South Korea accounted for 62% of the included studies. China accounted for 15% of the included publications. Publications also originated from the United Arab Emirates (5%) and the United States (4%).

**Figure 4:**
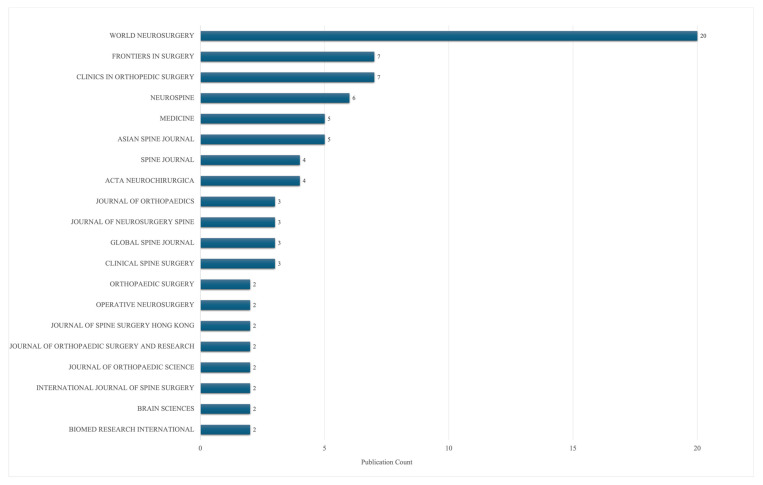
Distribution of included publications by journal.

Included publications were distributed across 34 journals (Figure 4). World Neurosurgery accounted for 20 included publications. This was followed by Clinics in Orthopedic Surgery and Frontiers in Surgery, with a record count of seven each.

**Figure 5:**
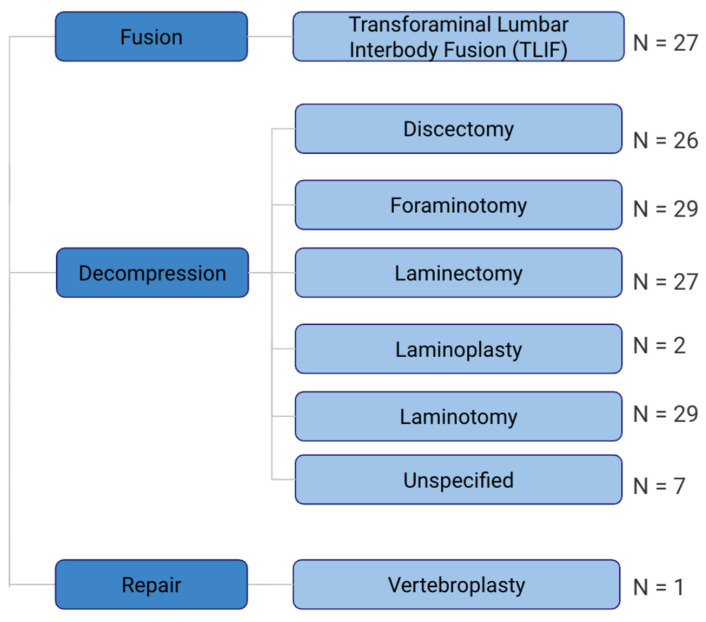
Technique applications of the included publications.

The spinal region addressed by each publication was recorded. Ninety-four publications addressed the lumbar spine (Table 3). Six publications addressed the cervical spine, and four addressed the thoracic spine. Three articles highlighted more than one region of the spine. Procedural applications reported in each publication are summarized in Figure 5. Several publications reported multiple procedural applications, resulting in 147 total procedural classifications. In addition, 27 articles discuss the application of biportal endoscopic spine surgery in transforaminal lumbar interbody fusion (TLIF). The most common way of decompressing the spine was conducting a foraminotomy and laminotomy (*N* = 29 each). This was followed by laminectomy (*N* = 27) and discectomy (*N* = 26). The least common technique for decompression was laminoplasty (*N* = 2). Seven articles did not specify how the spine was decompressed. Only one article discusses the use of repairing compression fractures with a biportal endoscopic technique (*N* = 1).

## 4. Discussion

As spine instrumentation and minimally invasive techniques have evolved over time, endoscopy has played a transformative role in improving visualization in tight working spaces [13]. Introduced about three decades ago, endoscopic visualization can reduce surgical dissection and tissue damage, length of hospital stay, rates of incidental durotomy, and decrease intraoperative blood loss [14,15,16,17]. More recently, the biportal endoscopic technique has been applied to the treatment of cervical, thoracic, and lumbar pathologies [8,18,19,20]. Relative to the uniportal approach, the biportal technique is thought to have a more manageable learning curve, widen the endoscopic view, and allow for greater instrument flexibility, notably with spinal canal decompression [10,12,21,22]. Given the rapidly growing interest in this technique, the current study aimed to synthesize influential publications to clarify how biportal endoscopic spine surgery has evolved in terms of indications, procedural applications, and clinical focus. By pairing citation-informed identification of influential studies with manual classification of procedural applications and anatomic focus, we provide a focused map of how the biportal technique has been studied and disseminated. Interpretations below are framed in the context of observed publication patterns rather than as definitive comparative outcome conclusions.

We observed that influential publications on biportal endoscopic spine surgery increased markedly after 2020, with peak publication activity occurring in 2022. Citation counts ranged from 5 to 297. Prior work has similarly demonstrated substantial growth in the endoscopic spine surgery literature over time [23]. The biportal technique was most commonly discussed in reference to foraminotomy, laminotomy, laminectomy, and discectomy, reflecting the widespread application of the biportal technique to decompressive procedures. In addition, a substantial proportion of influential publications addressed the application of biportal techniques to transforaminal lumbar interbody fusion (TLIF). Early landmark publications focused on posterolateral endoscopic excision of lumbar disc herniations, reflecting the initial clinical emphasis of the technique [24]. Prior reviews of the endoscopic spine surgery literature have identified earlier periods of rapid publication growth [25]. These findings highlight the relatively recent and rapid expansion of the biportal technique across multiple institutions worldwide. A contributing factor to its rapid implementation may be its learning curve; it has been noted that the biportal technique offers more familiar hand movements and flexible device handling relative to uniportal endoscopy, which has a more limited working field [26]. The technique is also ergonomically aligned with arthroscopy of the knee, shoulder, and hip, which is familiar to many orthopedic surgeons.

Prior analyses of the endoscopic spine surgery literature have reported differing geographic patterns of publication depending on technique and time period [25]. When considering the broader endoscopic spine surgery literature, contributions from China, South Korea, and the United States have been particularly prominent [23]. In the present review, a majority of influential biportal endoscopic spine surgery publications originated from South Korea. This is consistent with South Korea being the country in which the “biportal” endoscopic technique was coined in 2016 and where the biportal endoscopic approach was rapidly applied to treat various spine pathologies [27]. During this innovative explosion in Korea, BE was implemented to treat a wide range of spine conditions, including cervical disc herniation and cervical stenosis, thoracic ossification of the ligamentum flavum, and lumbar disc herniation and stenosis [28,29,30,31,32]. The predominance of South Korean authors and institutions likely reflects the origin of the technique and concentration of early adopters rather than global clinical consensus. High citation counts in this context may be driven by local networks, frequent cross-citation among groups using similar techniques, and interest from surgeons exploring the approach, rather than by widespread implementation across diverse practice settings. Thus, our findings should not be interpreted as proof of universal clinical acceptance of biportal surgery, but rather as a map of where the technique has been most actively developed and studied.

With respect to publication venues, influential biportal endoscopic spine surgery studies were most frequently published in World Neurosurgery. This is unsurprising, as prior studies have demonstrated that World Neurosurgery has the highest number of spine publications [33,34]. Several influential publications were authored by groups closely involved in the early development of the biportal technique, including work led by Dae-Jung (DJ) Choi. Choi formally invented biportal endoscopic spine surgery for recurrent disc herniations in South Korea in 2016 and has been one of the most influential figures in its growing application to the treatment of spinal pathologies [35].

Notably, the influential literature on biportal endoscopic spine surgery has focused predominantly on the lumbar spine. There are several plausible reasons behind the primary application of the biportal technique to the lumbar spine. First, endoscopy is a saline-based method, and there is a concern that water pressure may cause the epidural pressure to rise and disrupt the flow of CSF, contributing to postoperative complications such as seizure [36]. However, studies on lumbar spine endoscopy have shown that water pressure during endoscopic surgery is safe as long as irrigation pressure is maintained with appropriate technique and equipment, such as Luer lock stopcocks and outflow cannulas [37,38]. Still, there remains a gap in the literature on the safety and optimal ranges of water pressure in cervical and thoracic regions. As the cervical and thoracic spines include the spinal cord, concerns about neurologic injury and safety may have led to the initial application of BE in the lumbar region. Secondly, cervical pathology has traditionally been approached anteriorly because the spinal cord limits safe posterior retraction, making anterior cervical discectomy and fusion (ACDF) and cervical disc arthroplasty (CDA) well-established options for decompression [39,40]. The historical reliance on anterior approaches may partially explain the lower representation of cervical applications within influential biportal publications. Nonetheless, biportal endoscopic cervical foraminotomy may have a future role in selected patients by allowing targeted nerve-root decompression while preserving motion and potentially avoiding fusion. Although further research is warranted, 6% and 4% of our top 100 studies applied the biportal endoscopic approach to the cervical and thoracic regions, respectively.

Previous reviews have examined the emerging literature on biportal endoscopic spine surgery [41]. These reviews similarly noted the concentration of early publications within South Korea and frequent publication in select spine journals. In the present review, we further describe the procedural contexts in which biportal techniques have been applied and highlight the historical predominance of lumbar indications.

On a final note, one critical consideration of the biportal endoscopic technique is its benefits and drawbacks compared to other techniques such as uniportal spine endoscopy. While it may be easier to access the contralateral foramen by using a smaller uniportal endoscope, the biportal approach allows for triangulation of the instruments and scope and is thus associated with a wider visual field, greater maneuverability, and potentially easier learning curve due to instrument and technique familiarity [42]. On the other hand, the increased number of instruments may complicate the targeting of specific pathology and accessing the contralateral foramen. Furthermore, increased bleeding leading to “red screen” may be a cause of increased operative times even after the initial learning curve [43]. Additionally, the uniportal approach may be more difficult for the spine surgeon to master due to limitations in motion from using one working channel, a narrow area of vision (must rotate endoscope for adequate visualization), and poorer familiarity [42]. Future research is necessary to further elucidate ever-changing trends in biportal endoscopy, including its increasing adoption in the United States as well as its relevant technical considerations, advantages, and outcomes relative to other spinal endoscopy techniques.

## 5. Limitations

There are several limitations to consider in this review. First, although the literature search was conducted on 9 July 2025, the citation-based inclusion approach inherently favors older publications that have had sufficient time to accrue citations. As a result, recently published studies, even if methodologically rigorous or clinically important, may be underrepresented in this analysis. Second, inclusion was limited to studies published in English or with an available English translation. Third, the search was conducted using a single citation-indexed database, which may not capture all relevant publications despite its broad coverage. In addition, citation-based selection inherently favors older publications and more prolific research groups, and citation counts do not equate to methodological rigor, level of evidence, or clinical effectiveness. This review did not perform a formal quality appraisal or comparative outcome synthesis, as the intent was to characterize patterns of influence and procedural application rather than to grade evidence strength. Accordingly, findings should be interpreted as a descriptive mapping of the influential literature rather than a prescriptive evaluation of clinical superiority.

## 6. Conclusions

The landscape of spine surgery has been transformed by minimally invasive techniques, including endoscopic spine surgery. This review highlights the rapid clinical adoption of biportal endoscopic spine surgery, with the influential literature reflecting predominant application to the lumbar spine and early development centered in South Korea. Future research should focus on comparative outcomes, learning curves, and broader adoption of biportal endoscopic techniques across diverse practice settings.

## Figures and Tables

**Table 1:** The most-cited articles in biportal endoscopic spine surgery.

Rank	Title	Authors	Total Citations	Average Citations per Year
1	Fully endoscopic lumbar interbody fusion using a percutaneous unilateral biportal endoscopic technique: technical note and preliminary clinical results	Heo, D. H.	197	24.63
2	Percutaneous biportal endoscopic decompression for lumbar spinal stenosis: a technical note and preliminary clinical results	Eum, J. H.	162	18
3	Learning Curve Associated with Complications in Biportal Endoscopic Spinal Surgery: Challenges and Strategies.	Choi, D.	113	12.56
4	Clinical comparison of unilateral biportal endoscopic technique versus open microdiscectomy for single-level lumbar discectomy: a multicenter, retrospective analysis	Kim, S.	111	15.86
5	Endoscopic transforaminal lumbar interbody fusion: a comprehensive review	Ahn, Y.	102	17
6	Biportal Endoscopic Transforaminal Lumbar Interbody Fusion with Arthroscopy.	Kim, J.	95	13.57
7	A Systematic Review of Unilateral Biportal Endoscopic Spinal Surgery: Preliminary Clinical Results and Complications	Lin, G.	93	15.5
8	Biportal Endoscopic Spinal Surgery for Lumbar Spinal Stenosis	Kim, J.	87	14.5
9	Biportal endoscopic versus microscopic lumbar decompressive laminectomy in patients with spinal stenosis: a randomized controlled trial	Park, S.	86	17.2
10	How I do it? Biportal endoscopic spinal surgery (BESS) for treatment of lumbar spinal stenosis	Choi, C. M.	86	9.56

**Table 2:** Institutions affiliated with 5 or more articles in the most influential articles.

Affiliations	Article Count
Yonsei University	12
Chungnam National University	11
Barun Hospital	11
Hallym University	9
Himnaera Hospital	9
Leon Wiltse Memorial Hospital	9
Himchan Hospital	8
Korea University	8
Andong Hospital	8
Seoul Bumin Hospital	8
Seoul National University	6
University Hospital Sharjah	6
Catholic University of Korea	5
Kyungpook National University	5

**Table 3:** Distribution of biportal endoscopic spine surgery publications by spinal region.

Spine Region	Count
Cervical	6
Thoracic	4
Lumbar	94

## Data Availability

The original contributions presented in the study are included in the article, further inquiries can be directed to the corresponding author.
